# A universal point-of-care immunochromatographic test for the serodiagnosis of hepatitis D

**DOI:** 10.1128/jcm.01999-24

**Published:** 2025-04-11

**Authors:** Thiciany Blener Lopes, Fabiana Fioravante Coelho, Tárcio Peixoto Roca, Jéssica Karoline Augusta Oliveira, Valérian Delagarde, Ségolène Brichler, Diana Paola Gómez Mendoza, Juan Miguel Villalobos Salcedo, Deusilene Souza Vieira, Frédéric Le Gal, Ricardo Tostes Gazzinelli, Ana Paula Fernandes

**Affiliations:** 1 Department of Biochemistry & Immunology, Institute of Biological Sciences, Universidade Federal de Minas Gerais 28114, Belo Horizonte, Brazil; 2 Centro de Tecnologia em Vacinas, Universidade Federal de Minas Gerais 28114, Belo Horizonte, Brazil; 3 Faculty of Pharmacy, Universidade Federal de Minas Gerais 28114, Belo Horizonte, Brazil; 4 Laboratory of Molecular Virology, Fundação Oswaldo Cruz de Rondônia, Porto Velho, State of Rondônia, Brazil; 5 French National Reference Center for Hepatitis B, C and D Viruses, Laboratoire de Microbiologie Clinique, Hôpital Avicenne 55069, Bobigny, France; 6 Instituto René Rachou, Fundação Oswaldo Cruz-Minas, Belo Horizonte, Brazil; Theel, Mayo Clinic Minnesota, Rochester, Minnesota, USA

**Keywords:** Immunochromatographic test, hepatitis D, hepatitis B, serological diagnosis, HDV recombinant antigen, HDV genotyping

## Abstract

Hepatitis D is estimated to affect 12 million people worldwide and is caused by the hepatitis D virus (HDV), a defective virus that requires the presence of the hepatitis B virus (HBV) for infection. Here, we report a new recombinant antigen (DTH10.1) to detect anti-HDV IgG antibodies, designed to include a consensus sequence of the HDV antigen, based on bioinformatic analysis of the eight HDV genotypes. Using serum samples from patients living in the endemic area of the Brazilian Amazon basin, the enzyme-linked immunosorbent assay (ELISA) based on this protein displayed a sensitivity of 90.6% (95% CI: 84.2%–94.5%) and a specificity of 100% (95% CI: 94.2%–100.0%), while a rapid immunochromatographic test (ICT) showed a sensitivity of 91.3% (95% CI: 85.0%–95.1%) and specificity of 99.0% (95% CI: 94.5%–99.95%). Commercial monoclonal antibodies for HBV surface antigen (HBsAg) detection were then added to the test, resulting in a multiplex ICT with a sensitivity of 87.1% (95% CI: 81.3%–91.4%) for HBsAg and 95.2% (95% CI: 90.0%–97.8%) for anti-HDV IgG and specificity of 100% (95% CI: 91.0%–100.0%) and 98.0% (95% CI: 92.9%–99.6%), respectively. Finally, the three tests were evaluated against a panel of 79 patient samples infected, covering the eight HDV genotypes. The results indicated that all the DTH10.1-based tests were able to detect anti-IgG HDV antibodies with high sensitivity and specificity, regardless of the infecting HDV genotype. In conclusion, the prototypes developed for serodiagnosis of HDV using the DTH10.1 recombinant protein are promising tools for the universal diagnosis of HDV infection.

**IMPORTANCE:**

The manuscript outlines the complete strategy for developing tools for the diagnosis of hepatitis D, including an enzyme-linked immunosorbent assay (ELISA), an immunochromatographic test (ICT), and a multiplex ICT for the simultaneous detection of hepatitis B virus surface antigen and anti-hepatitis D virus (HDV) IgG antibodies. All the tests described are capable of detecting all eight HDV genotypes with high accuracy.

## INTRODUCTION

Hepatitis D is an infectious disease caused by the hepatitis D virus (HDV), a satellite RNA virus that depends on the hepatitis B virus (HBV) for the formation of the viral envelope and entry into hepatocytes (1, 2). Worldwide, HDV affects an estimated 12 million people (3) with prevalence varying across regions, reaching up to 50% among HBV-positive patients in parts of central Africa (4), central Asia (5), and some areas of the Amazon basin (6, 7). The HDV belongs to the *Deltavirus* genus and the *Kolmioviridae* family, with eight distinct genotypes (HDV-1 to HDV-8) (8).

Hepatitis D may occur as a superinfection when individuals with chronic hepatitis B are infected with HDV, or as a simultaneous co-infection with both viruses (3). The progression of hepatitis is three times higher in HDV/HBV infections compared to HBV alone, with an increased risk of developing liver cancer and cirrhosis (9). Cirrhosis evolution is observed in 10% to 15% of patients within 2 years and in 70% to 80% of patients within 5–10 years after infection (10, 11).

Chronic hepatitis B infection is defined by the persistent detection of HBV surface antigen (HBsAg) in plasma. HBV screening is carried out through the detection of HBsAg as it is a hallmark of the hepatitis B infection (12, 13), although the analytical sensitivity of HBsAg assays may depend on virus genotype or subtype and HBV genome recombination events that may lead to changes in HBsAg recognition (14, 15). Moreover, low levels of HBsAg may be undetected, leading to false-negative results (16).

For HBsAg-positive patients, HDV is diagnosed through serological and molecular tests, typically performed on individuals with suspected hepatitis D infection and in individuals living in or with travel history to endemic areas. In contrast to this testing pattern, it is crucial to screen all HBsAg-positive patients for HDV, as hepatitis D infection is widely underdiagnosed (17, 18). To emphasize this issue, the new World Health Organization (WHO) guidelines recommend universal HDV antibody testing for individuals with chronic hepatitis B and advocate for a two-step double reflex testing protocol after any positive HBsAg result: first, testing for anti-HDV antibodies, followed by HDV RNA testing in case of a positive result (19). This approach may help estimate the true epidemiological landscape of HDV infection, leading to more effective control measures and earlier therapeutic interventions, potentially preventing the characteristic liver complications associated with hepatitis D.

HDV diagnosis is based on total (IgG and IgM) HDV antibody screening, typically using enzyme-linked immunosorbent assay (ELISA) or chemiluminescence immunoassay (CLIA). However, these tests require complex laboratory infrastructure and trained personnel (20), making them largely inaccessible in lower- to middle-income countries, where the disease is often more prevalent. In this context, immunochromatographic tests (ICT) are more suitable for diagnosing HDV infection, as they are easy to transport and administer in field settings, highly stable, and provide rapid results to guide clinical and therapeutic decisions (21). Given the worldwide distribution and the genetic variability of HDV, these tests should display high sensitivity to detect infection across all eight HDV genotypes.

Here, we report the design and production of a recombinant protein based on pan-genotypic HDV antigen (HDAg) sequences. This protein was used to develop three serological assays for the detection of anti-HDV IgG antibodies: an ELISA and two ICTs, including one designed to detect both HBsAg and anti-HDV IgG simultaneously.

## MATERIALS AND METHODS

### Human serum samples

**Fig 1:**
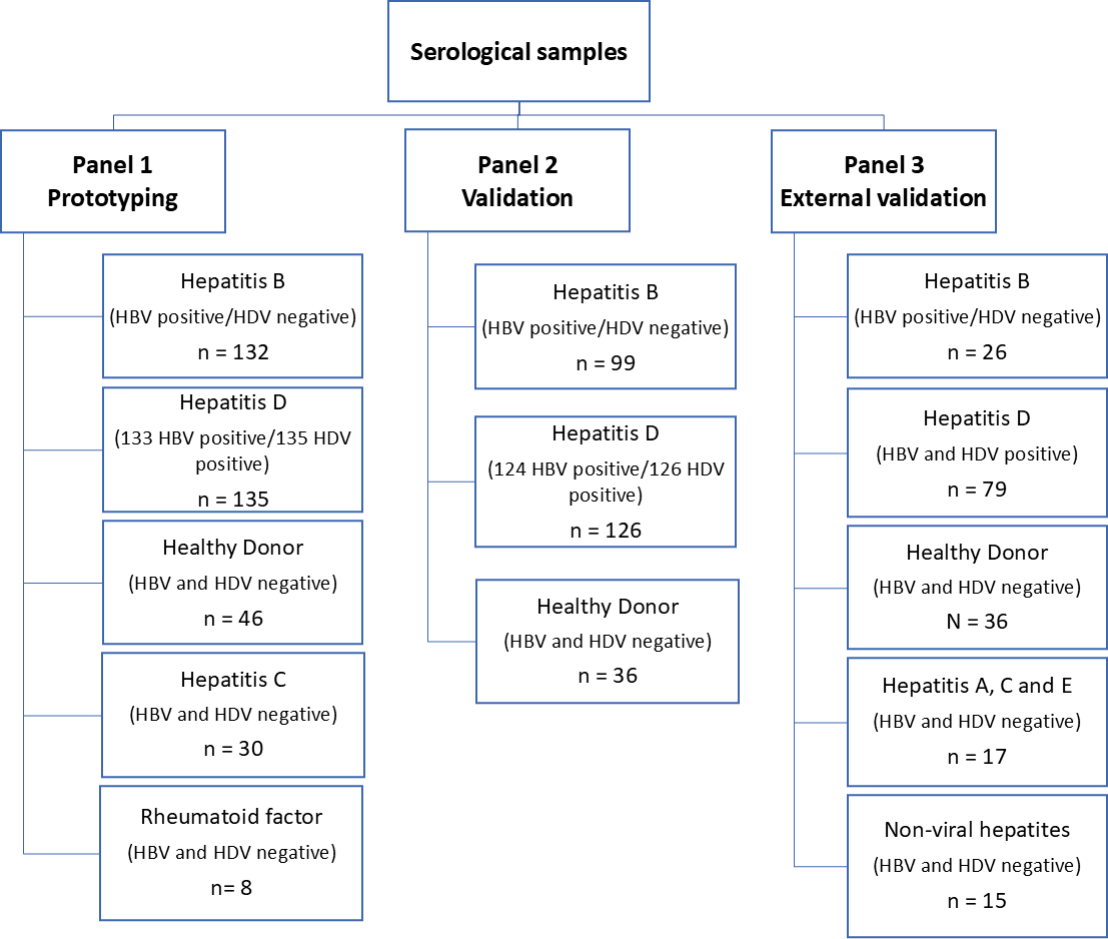
Panels of samples for the prototyping and validation steps of the developed tests. All hepatitis samples from panels 1 and 2 were tested with Diasorin ETI-AB-DELTAK-2 at LACEN of Rondônia and had their results confirmed at CT-Vacinas (Belo Horizonte, Brazil) by either GB HDV Ab kit (panel 1) or ELISA HDV Ab Dia.Pro (panel 2). The samples from panel 3 were tested with Diasorin Liaison XL Murex Anti-HDV.

    Samples were distributed into three panels (Fig. 1), with each of their characteristics described in Table 1. Panel 1, which was used for the prototyping and development of the tests, consisted of samples from healthy subjects (*n* = 46), negative for hepatitis B and D in commercial ELISA (BIOELISA HBsAg by Bioclin and ELISA HDV Ab Dia.Pro), respectively, and HBsAg-positive samples (*n* = 20) from people living in Belo Horizonte (Minas Gerais, non-endemic state); HBsAg-positive samples (*n* = 112) and hepatitis D samples (*n* = 135) from individuals of the north region of Brazil (HDV endemic area) diagnosed by Central Laboratory of Rondônia (LACEN) using routine diagnostic tests (Diasorin ETI-AB-DELTAK-2 ELISA for hepatitis D samples); samples with altered levels of rheumatoid factor (*n* = 8) and hepatitis C-positive (*n* = 30) were used for cross-reactivity testing. The results of 78 hepatitis D-positive samples were confirmed using the GB HDV Ab ELISA kit, a commercial test by General Biological Corporation (Taiwan) for anti-HDV detection.

**TABLE 1:** Characteristics of serum samples used during the prototyping and validation of the developed tests

Group	Origin of samples	*N*	HBV status	HDV status	Other diagnoses
Healthy (negative) donors	Non-endemic areas, Brazil	82	All negative for HBsAg	All negative for anti-HDV antibodies (ELISA HDV Ab Dia.Pro)	ND^*a*^
Hepatitis B	Endemic areas of Brazil. 95.14% from Rondônia state	203	All samples positive for HBsAg, 10.5% for HBeAg^*b*^, 89.5% for anti-HBe^*b*^, 99.0% for total anti-HBc^*c*^. All but one were negative for anti-HBs^*d*^. 35.9% under treatment	All negative (Diasorin ETI-AB-DELTAK-2)	Negative for HIV infection
Hepatitis B	Non-endemic areas, Brazil	20	All positive for HBsAg (HBsAg Assay by Siemens Healthineers) Not tested for other HBV markers	All negative (ELISA HDV Ab Dia.Pro)	ND^*a*^
Hepatitis B	French National Reference Center for Hepatitis Delta	26	All samples positive for HBsAg	All negative (Diasorin Liaison XL murex Anti-HDV)	ND^*a*^
Hepatitis D	Endemic areas of Brazil. 91.4% from Rondônia State	268	264 samples positive for HBsAg and 6.25% for anti-HBs, 6.25% for HBeAg, 91.7% for anti-HBe and 87.5% for total anti-HBc. 4 samples negative for HBsAg and positive for anti-HBs and anti-HBc or anti-HBe.	All positive (Diasorin ETI-AB-DELTAK-2) 126 samples were retested (ELISA HDV Ab Dia.Pro)	Negative for HIV infection
Hepatitis D	French National Reference Center for Hepatitis Delta	79	All samples positive for HBsAg	All positive (Diasorin Liaison XL murex Anti-HDV). HDV-1 non-African (*n* = 12.7%), HDV-1 African (*n* = 21.5%), HDV-2 (*n* = 3.8%), HDV-3 (*n* = 2.5%), HDV-4 (*n* = 7.6%), HDV-5 (*n* = 19%), HDV-6 (*n* = 11.4%), HDV-7 (*n* = 10.1%), HDV-8 (*n* = 8.9%), Unknown (2.5%)	ND^*a*^
Hepatitis C	Endemic areas, northern region of Brazil	30	All negative for the main HBV serological markers	All negative for anti-HDV antibodies	Negative for HIV infection. Positive in serology for hepatitis C infection
Hepatitis C	French National Reference Center for Hepatitis Delta	6	All negative for the main HBV serological markers	All negative (Liaison XL murex Anti-HDV)	Positive for HCV antibodies
Hepatitis A	French National Reference Center for Hepatitis Delta	6	All negative for the main HBV serological markers	All negative (Liaison XL murex Anti-HDV)	Positive for HAV IgM antibodies
Hepatitis E	French National Reference Center for Hepatitis Delta	5	All negative for the main HBV serological markers	All negative (Liaison XL murex Anti-HDV)	Positive for HEV-RNA
Biliary hepatitis	French National Reference Center for Hepatitis Delta	5	All negative for the main HBV serological markers	All negative (Liaison XL murex Anti-HDV)	Negative for viral hepatitis
Autoimmune hepatitis	French National Reference Center for Hepatitis Delta	5	All negative for the main HBV serological markers	All negative (Liaison XL murex Anti-HDV)	Negative for viral hepatitis
Alcoholic hepatitis	French National Reference Center for Hepatitis Delta	5	All negative for the main HBV serological markers	All negative (Liaison XL murex Anti-HDV)	Negative for viral hepatitis
Rheumatoid factor	Non-endemic areas, Brazil	8	All negative for HBsAg	All negative for anti-HDV antibodies	Samples with rheumatoid factor varying between 18.2 to 280 UI/mL

^*a*^ ND, no data.

^*b*^ HBeAg or HBe: Hepatitis B "e" antigen.

^*c*^ anti-HBc: Hepatitis B core antibody.

^*d*^ anti-HBs: Hepatitis B surface antibody.

Samples composing panel 2 were selected randomly from the sample collection of the Laboratório de Virologia Molecular–Fundação Oswaldo Cruz Rondônia (FIOCRUZ-RO) to validate the prototypes of the developed tests. They consisted of 225 samples positive for hepatitis, of which 98 were considered positive only for HBsAg, 124 positive for both anti-HDV antibodies and HBsAg, 2 positives only for anti-HDV antibodies, and 1 positive for anti-HBs (hepatitis B surface antibody) due to previous infection (anti-HBe positive). Additionally, 36 samples from healthy donors composed this panel. Among the hepatitis D samples (*n* = 126), 22.2% (*n* = 28) had detectable viral RNA, 21.4% (*n* = 27) were negative, and 56.4% (*n* = 71) did not have available data. All these samples were tested using the Diasorin ETI-AB-DELTAK-2 at LACEN of Rondônia and later were retested using the ELISA HDV Ab Dia.Pro at CT-Vacinas (Belo Horizonte, MG) to confirm the results.

Overall, panels 1 and 2 included 268 positive samples for hepatitis D, of which 264 were also positive for HBsAg and 4 were negative. The HBsAg-positive samples (*n* = 264) were also tested for other HBV markers, and 6.25% of them were positive for anti-HBs, 6.25% were positive for HBeAg (hepatitis B "e" antigen), 91.7% for anti-HBe, and 87.5% for total anti-HBc (hepatitis B core antibody). The four samples negative for HBsAg were also tested for the other markers and were positive for anti-HBc or anti-HBe and positive for anti-HBs. Considering the results of the additional HBV markers, these patients were exposed to HBV infection, but had no active HBV infection at the time of sample collection. Therefore, they were maintained in our study as positive samples for both HBV and HDV infection.

Panel 3 consisted of 137 samples belonging to the sample collection of the Laboratoire de Microbiologie Clinique, Hôpital Avicenne, which is the French National Reference Center for Hepatitis Delta. These samples are positive for hepatitis B (*n* = 26), hepatitis D (*n* = 79), hepatitis C (*n* = 6), hepatitis A (*n* = 6), hepatitis E (*n* = 5), biliary hepatitis (*n* = 5), alcoholic hepatitis (*n* = 5), and autoimmune hepatitis (*n* = 5). The hepatitis D samples were all tested using the Diasorin Liaison XL murex Anti-HDV. Among these samples, 46.8% (*n* = 37) were positive for HDV RNA and 53.2% (*n* = 42) were negative. All HDV positive samples were genotyped and included 10 non-African HDV-1 samples, 17 African HDV-1 samples, 3 HDV-2, 2 HDV-3, 6 HDV-4, 15 HDV-5, 9 HDV-6, 8 HDV-7, and 7 HDV-8 samples. HDV genotype was determined as described by Ivaniushina et al. (22), by Sanger bidirectionally sequencing method of the « R0 » region of the genome located at the end of the HD coding gene.

### Expression and purification of the recombinant protein DTH10.1

A full-length alignment of 47 HDAg sequences covering the eight HDV genotypes available in the GenBank database was performed to generate a consensus sequence using the programs Protein BLAST and Multiple Align Show. The resulting sequence was modified to encode a recombinant protein named DTH10.1. This sequence was inserted into the pET-24a vector, and later it was subcloned, using restriction enzymes, into the commercial vector Champion pET SUMO (Invitrogen).

Competent *Escherichia coli* BL21(DE3) cells were transformed with either the pET24a/DTH10.1 or the pET SUMO/DTH10.1 for the expression of DTH10.1 or DTH10.1-SUMO proteins, respectively. The antigens were purified from soluble fractions by affinity chromatography using a Ni^2+^-charged column in an ÄKTA Prime Plus System (GE Healthcare, USA), according to the manufacturer’s instructions and analyzed by sodium dodecyl sulfate-polyacrylamide gel electrophoresis (SDS-PAGE). An anti-DTH10.1 polyclonal antibody was obtained from New Zealand rabbit immunized with the DTH10.1 recombinant protein. The peptides were confirmed by liquid chromatography tandem-mass spectrometry (LC-MS/MS), in an Orbitrap Exploris 240 mass spectrometer coupled to a Nano LC-MS/MS nanoflow chromatography system (Dionex ultimate 3000 RLSCnano System) (ThermoFisher Scientific, USA) and analyzed using Proteome Discoverer v.2.5.

### DTH10.1 ELISA

The prototyped ELISA consisted of high-binding plates of polystyrene coated with the DTH10.1 antigen, diluted in a carbonate buffer (pH 9.6) (100 µL/well), and blocked with bovine serum albumin. Serum samples were added to each well at a dilution of 1:100 in phosphate-buffered saline with Tween 20 (PBS-T) and incubated for 60 min at 37°C. Wash the plate with four cycles of wash buffer. The antibody-antigen binding was detected by the addition of peroxidase-conjugated anti-human IgG (Sigma-Aldrich) diluted in PBS-T and incubated for 45 min at 37°C, followed by another wash cycle and the addition of tetramethylbenzidine (Scienco) for 45 min at 37°C. The reactions were stopped using a 0.5 M H_2_SO_4_ solution. Plates were analyzed at an optical density of 450 nm using the Multiskan GO microplate spectrophotometer (ThermoFisher Scientific, USA). The cut-off points were set at three standard deviations above the mean optical density of the negative samples. The results were expressed as a reactivity index (RI), calculated by the ratio of sample absorbance over the cut-off value. RI values > 1.1 were considered positive, RI values < 0.9 were considered negative, and values in between were considered indeterminate and excluded from the analysis. The stability of the sensitized plates of DTH10.1 ELISA was accelerated by storage at 37°C to predict the shelf life of the kits. The performance was assessed at 7, 14, 21, and 28 days of storage, and the result was compared with those obtained from a control plate stored at 4°C.

### ICT

The control and test line components were applied to a nitrocellulose membrane and fixed onto an adhesive card using an automatic dispenser (Autokun). The HDV antigen or the HBV antibody were conjugated to colloidal gold nanoparticles (AuNP) and dispensed onto a fiberglass membrane. All the membranes were assembled, cut into strips, and inserted into a plastic cassette. The reading of results was set at 20 min after sample application, and any intensity of color observed in the test line is considered a positive result. Both positive and negative samples must show reactivity in the control line.

A rapid ICT for detecting anti-HDV IgG antibodies was assembled using the DTH10.1-SUMO to optimize protein conjugation to gold nanoparticles. Monoclonal anti-human IgG antibodies (Fapon Biotech) were immobilized on a nitrocellulose membrane to form the test line, and anti-rabbit IgG antibodies were immobilized to form the control line, both in concentrations varying between 0.2 and 5.0 mg/mL. The DTH10.1-SUMO protein and rabbit IgG were conjugated to AuNP.

In order to improve the diagnosis of hepatitis D in endemic areas, a multiplex ICT was designed, with the presence of two test lines, one for anti-HDV IgG and the other for HBsAg. To do so, a pair of monoclonal anti-HBs antibodies (mAb C and mAb J, Fapon Biotech) was used in addition to the components of the anti-HDV ICT. In test line 1, the mAb C, used to capture the HBsAg, were immobilized on the nitrocellulose membrane at a concentration between 0.2 and 5.0 mg/mL. Test line 2 (center line) consisted of the monoclonal anti-human IgG. The control line was designed using a commercial HBsAg (Fapon Biotech). The conjugate was composed of a mix of DTH10.1 and mAb J conjugated to AuNP at a 1:1 ratio.

The analytical sensitivity of the multiplex ICT for HBsAg was also evaluated using the WHO HBsAg standard (HBV genotype B4, National Institute for Biological Standard Control [NIBSC] code number 12/226) from the NIBSC. The standard was diluted in 0.85% saline solution at known concentrations ranging from 10 UI/mL to 3 UI/mL to determine the lowest concentration of antigen detected by the ICT.

### Statistical analysis

Statistical analysis was performed using GraphPad Prism (version 8.0). Sensitivity, specificity, accuracy, ROC (receiver operating characteristic) curve, and 95% confidence intervals (CIs) were calculated and compared to the reference data. The Mann-Whitney and chi-squared tests were used in a two-tailed analysis, and significant differences were considered when *P* < 0.05. The concordance between tests was calculated using the kappa (κ) index, according to Cohen (23), and interpreted according to Landis and Koch (24): 1.00–0.81 = excellent; 0.80–0.61 = good; 0.60–0.41 = moderate; 0.40–0.21 = weak; and 0.20–0.00 = negligible agreement.

## RESULTS

### Engineering the DTH10.1 protein

**Fig 2:**
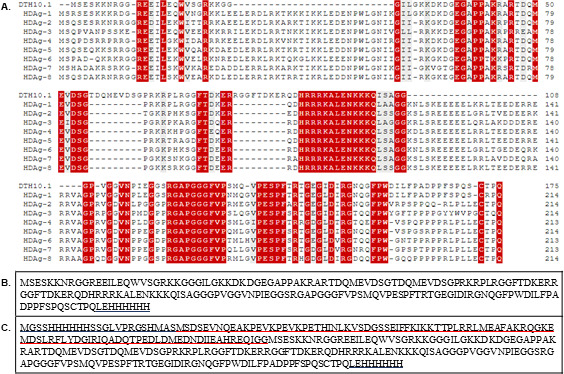
Amino acid sequence of the recombinant HDAg protein. (A) Amino acid sequence alignment of DTH10.1 and HDAg consensus sequences for genotypes 1–8. Consensus sequences were determined by a full-length alignment of 47 HDAg sequences covering the eight HDV genotypes available in the GenBank database. Regions marked in red represent consensus amino acids for all sequences. These are the most preserved regions among the different virus genotypes. (B) Sequence of DTH10.1 recombinant protein. (C) Sequence of DTH10.1-SUMO recombinant protein. The red underline indicates the N-terminal SUMO tag, and the black underline indicates non-HDAg amino acids, including the His-Tag (HHHHHH) sequence.

The alignment of the HDAg sequences covering the eight HDV genotypes resulted in a sequence that encoded a 19.9 kDa protein named DTH10.1 (Fig. 2A and B). To improve the physicochemical properties of DTH10.1, the protein-coding sequence was subcloned into the vector pET SUMO, generating a protein with approximately 33 kDa named DTH10.1-SUMO (Fig. 2C).

**Fig 3:**
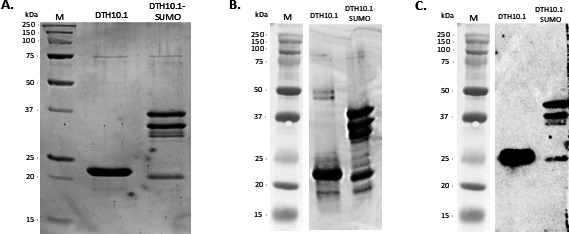
Recombinant DTH10.1 and DTH10.1-SUMO protein. (A) SDS-PAGE of the DTH10.1 and DTH10.1-SUMO recombinant proteins after purification by affinity chromatography. (B) Western blot of both proteins using a polyclonal antibody anti-DTH10.1. (C) Western blot of the proteins using an antibody to the histidine tag. Molecular weight markers (M) are shown on the left. The images of the molecular weight marker in panels B and C are duplicates of a single SDS-PAGE gel.

DTH10.1 and DTH10.1-SUMO were expressed as soluble proteins. DTH10.1 was expressed as a single band with an estimated molecular weight of 21.0 kDa (Fig. 3A), yielding 52 mg of protein per liter of culture. The purified DTH10.1-SUMO presented three main protein bands in SDS-PAGE: two of approximately 36 kDa and 33 kDa, compatible with the protein DTH10.1 plus the SUMO tag, which was expected to be 33 kDa, and other bands resulting from protein cleavage. The yield of this protein was 95 mg/L of culture. All observed fractions from the two proteins were recognized by anti-histidine and anti-DTH10.1 antibodies in Western blot analysis (Fig. 3B and C).

The LC-MS/MS analysis was performed on the bands observed on the SDS-PAGE gel for the DTH10.1 and the DTH10.1-SUMO proteins. All samples displayed similar peak patterns in the generated mass spectrum, with coverages ranging from 59.5% to 76.5% (Fig. S1 and S2), compared to the reference sequence of the DTH10.1 protein. The bands present in the DTH10.1-SUMO profile were recognized as the HDAg, suggesting that the different bands may have been generated by the degradation of the SUMO tag.

### DTH10.1 ELISA

**Fig 4:**
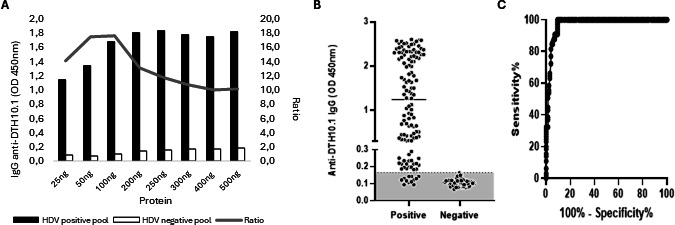
Evaluation of the ability of recombinant protein DTH10.1 to distinguish positive from negative samples by ELISA. (A) Titration of DTH10.1 protein for setting the amount of antigen to coat the ELISA plates. Different antigen titers were analyzed, starting from 25 ng and increasing until 500 ng. The sera pool samples were diluted 1:100. The gray line indicates the ratio obtained by dividing the average absorbance of the positive samples by the negative samples. (B) Distribution chart of the levels of anti-HDAg antibodies in positive (*n* = 127) and negative samples (*n* = 62) of panel 1. ELISA was performed with 100 ng of DTH10.1 and sera dilutions of 1:100. The gray area represents absorbance below the cut-off value, which was 0.166. (C) Area of the ROC curve constructed from the absorbance values of all tested samples. The area under the curve value is 0.9735 with a *P*-value <0.0001.

The first analysis aimed at defining the ELISA conditions using the DTH10.1 protein. To determine the appropriate antigen quantity for coating the ELISA plate, protein amounts ranging from 25 ng to 500 ng per well were tested (Fig. 4A). Based on the absorbance readings of the positive and negative serum pools (diluted 1:100 in PBS-T buffer) and the resulting ratio (positive/negative absorbance values), the optimal results were obtained with 100 ng of protein, which was selected for coating the plates.

The DTH10.1 ELISA was evaluated with 189 individual samples (127 positive and 62 negative, from panel 1). In this assay, 12 of the positive samples did not react to DTH10.1, which corresponds to a sensitivity of 90.6% (95% CI: 84.2%–94.5%), while none of the negative samples reacted with the protein, resulting in a specificity of 100.0% (95% CI: 94.2%–100.0%). Three negative samples showed indeterminate results and were not included in the analysis. The accuracy of the test was 93.65% (95% CI: 89.2%–96.3%). This result demonstrated that DTH10.1 ELISA effectively differentiates between positive and negative HDV samples (Fig. 4B and C).

Next, the commercial GB HDV Ab kit, which requires 100 µL of undiluted samples, was used to confirm the current diagnostic results for 78 out of the 127 positive hepatitis D samples from panel 1. Of these, 73 samples tested positive and 5 tested negative, 2 of which were also negative in the DTH10.1 ELISA, conducted with a 1:100 serum dilution. Thus, when considering only positive samples in the two commercial assays, the sensitivity of the DTH10.1 ELISA would correspond to 91.8%.

Hepatitis B and C samples, along with samples containing rheumatoid factors, were tested for potential cross-reactivity in DTH10.1 ELISA, and none showed any reaction in the test. The accelerated stability was performed by comparing the plates stored at 37°C and 4°C for up to 28 days. The sensitized plates remained stable throughout the entire period, maintaining similar performance under both conditions (Table S1), predicting stability for at least 18 months under refrigerated conditions, which corresponds to the shelf life period of most ELISA kits (25).

### Single immunochromatographic test for anti-HDV IgG

**Fig 5:**
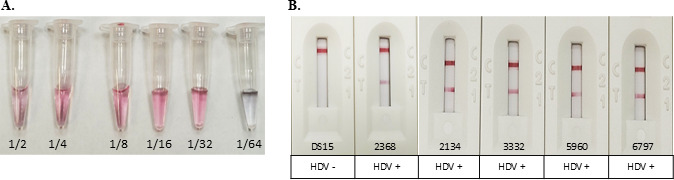
Development of single ICTs for anti-HDV IgG. (A) Illustrative results for titration of DTH10.1-SUMO with AuNP in different proportions of protein/AuNP (1/2 to 1/64) for conjugation. The pink color is indicative of a stable proportion for use, with the last pink one being the most recommended. The gray color is indicative of an unstable conjugation. (B) Evaluation of negative and positive samples with different antibody titers in ICT anti-HDV IgG. ELISA reactivity index for positive samples 2368, 2134, 3332, 5960, and 6797 are 8.8, 8.9, 7.6, 10.8, and 8.0, respectively. DS15 is a negative sample. Below the images are the expected results for each sample evaluated.

To develop the anti-HDV IgG ICT, the DTH10.1-SUMO protein was conjugated with AuNP. The aspects of the DTH10.1-SUMO-AuNP conjugation are illustrated in Fig. 5A and were sufficiently stable for ICT prototyping. Results from the ICT developed with positive samples, showing varying signal intensities for hepatitis D, are presented in Fig. 5B. To detect anti-HDV IgG, the specific antibodies present in the samples react with the DTH10.1-SUMO conjugated and migrate until they reach the test line, where they are retained, forming a reactive line.

The anti-HDV IgG rapid ICT development was performed using 10 µL of serum from 232 samples of panel 1 (63 hepatitis D samples, 123 hepatitis B samples, and 46 samples from healthy donors), previously tested with the DH10.1 ELISA. The sensitivity, specificity, and accuracy values were 82.5% (95% CI: 71.4%–89.96%), 92.9% (95% CI: 88.0%–95.9%), and 90.1% (95% CI: 85.6%–93.3%), respectively, compared with the Diasorin’s ELISA results.

A second ICT prototype, optimized for increased sensitivity, was evaluated using panel 2 (261 samples). This prototype successfully detected 115 out of 126 samples positive for anti-HDV antibodies. No reaction was observed in 98 out of the 99 hepatitis B samples and in all 36 healthy donor samples, resulting in a sensitivity of 91.3% (95% CI: 85.0%–95.1%), a specificity of 99.3% (95% CI: 95.2%–99.96%), an accuracy of 95.4% (95% CI: 92.1%–97.4%), and a kappa value of 0.91 for the ICT, using the ELISA HDV Ab Dia.Pro as standard (Table 2).

**TABLE 2:** Sensitivity, specificity, and accuracy of the ICTs developed using samples from panel 2

ICT	Sera samples^*a*^		
	Sensitivity 95% CI	Specificity 95% CI	Accuracy 95% CI
Anti-HDV IgG	91.3% 85.0%–95.1%	99.3% 95.2%–99.96%	95.4% 92.1%–97.4%
Multiplex anti-HDV IgG	95.2% 90.0%–97.8%	97.8% 93.7%–99.4%	96.6% 93.6%–98.2%
Multiplex HBsAg	87.1% 81.3%–91.4%	100.0% 91.0%–100.0%	89.4% 84.4%–92.9%

^*a*^ HBV- and HDV-negative samples: *n* = 36; HBV-positive and HDV-negative samples: *n* = 99; HBV- and HDV-positive samples: *n* = 124; HDV-positive and HBV-negative samples: *n* = 2. A total of 261 samples were tested. Tests were performed with 10 µL of sera samples in the anti-HDV IgG ICT and 50 µL in the multiplex ICT.

### Multiplex ICT: anti-HDV IgG and HBsAg

**Fig 6:**
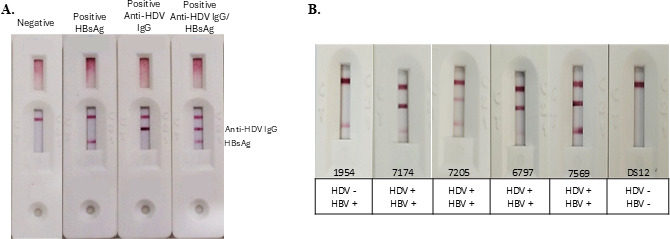
Development of the multiplex ICT. (A) Illustrative results of the multiplex ICT when samples negative, positive only for HBsAg, positive only for anti-HDV IgG, or positive for both markers are tested. The first test line (bottom to top) is for HBsAg, and the middle one is for anti-HDV IgG antibody detection. The top line corresponds to the test control. (B) Evaluation of of negative and positive samples with different reactivity for anti-HDV IgG and HBsAg. Sample 1954 is positive only for HBsAg, samples 7174, 7205, 6797, and 7569 are positive for both markers of infection, and DS12 is a negative sample. Below the images are the expected results for each sample evaluated.

When positive samples for both infections are assayed, HBsAg reacts with mAb J conjugated to AuNP, which is retained by mAb C at test line 1. Anti-HDV IgG antibodies bind to DTH10.1-SUMO conjugated to AuNP and migrate to test line 2, where they are retained. Therefore, the test demonstrates reactivity at both test lines. Samples from HBV-only infected patients react only at line 1, while samples from individuals who have recovered from both infections but still have circulating anti-HDV antibodies react in test line 2. Figure 6A and B illustrate the performance of the multiplex ICT.

The multiplex ICT was initially evaluated with 10 µL of samples from panel 1, resulting in a sensitivity of 84.1% (95% CI: 73.2%–91.1%), specificity of 91.1% (95% CI: 85.9%–94.6%), and accuracy of 89.2% (95% CI: 84.6%–92.6%) for anti-HDV IgG detection and 84.1% (95% CI: 78.1%–88.7%), 100.0% (95% CI: 92.9%–100.0%), and 87.5% (95% CI: 82.6%–91.2%), respectively, for the detection of HBsAg.

To increase the sensitivity of the ICT, panel 2 samples were subsequently tested using 50 µL volume. The ICT successfully detected 120 out of 126 anti-HDV positive samples, with no reaction observed in 97 out of 99 hepatitis B samples and in 35 out of the 36 healthy donor samples, resulting in a sensitivity value of 95.2% (95% CI: 90.0%–97.8%), a specificity of 97.8% (95% CI: 93.7%–99.4%), and an accuracy of 96.6% (95% CI: 93.6%–98.2%), using the ELISA HDV Ab Dia.Pro as standard. For HBsAg detection, the multiplex ICT showed 87.1% sensitivity (95% CI: 81.3%–91.4%), a specificity of 100.0% (95% CI: 91.0%–100.0%), and an accuracy of 89.4% (95% CI: 84.4%–92.9%), as shown in Table 2. The correlation results indicated a kappa value of 0.93 and 0.7 for anti-HDV IgG and HBsAg, respectively.

The analytical sensitivity of the multiplex ICT for the detection of HBsAg, using an international standard, was 5 IU/mL (Table S2).

### Evaluation of the ICTs with hepatitis D samples from the eight genotypes

To determine whether the tests would detect infections from HDV genotypes other than HDV-3, which is prevalent in the Brazilian Amazon Basin, the two ICTs and the ELISA were sent to the French National Reference Center for Hepatitis Delta and evaluated with samples from panel 3. As shown in Table 3, the tests demonstrated high sensitivity in detecting anti-HDV antibodies in samples from patients infected with any of the eight HDV genotypes, including HDV-1 samples from various regions. Out of 79 samples, 6 were not recognized by the anti-HDV IgG ICT, 3 samples by the multiplex ICT, and 3 by the ELISA. These samples belonged to genotypes 1, 5, and 8 or a nondetermined genotype. Across panel 3, the ELISA demonstrated a sensitivity of 98.7% (95% CI: 93.0%–99.9%), a specificity of 100.0% (95% CI: 84.5%–100.0%), and accuracy of 99.0% (95% CI: 94.4%–99.95%), respectively. The sensitivity, specificity, and accuracy for the anti-HDV IgG ICT were 92.4% (95% CI: 84.4%–96.5%), 90.5% (95% CI: 71.1%–98.3%), and 92.0% (95% CI: 85.0%–95.9%), respectively, and 96.2% (95% CI: 89.3%–99.0%), 81.0% (95% CI: 60.0%–92.3%), and 92.9% (95% CI: 86.1%–96.5%), respectively, for the multiplex ICT (anti-HDV IgG detection). Furthermore, the three tests achieved 100.0% sensitivity in samples with detectable HDV RNA. Comparing these results to the Liaison XL murex anti-HDV assay as the standard indicated a kappa value of 0.97 for the ELISA, 0.77 for anti-HDV IgG ICT, and 0.78 for the Multiplex ICT.

**TABLE 3:** Evaluation of the ICTs with hepatitis D samples from the eight HDV genotypes

HDV genotype	Anti-HDV ICT^*a*^	Multiplex ICT^*a*^	ELISA
HDV-1 non-African (*n* = 10)	10	10	9
HDV-1 African (*n* = 17)	17	17	17
HDV-2 (*n* = 3)	3	3	3
HDV-3 (*n* = 2)	2	2	2
HDV-4 (*n* = 6)	6	5^*b*^	6
HDV-5 (*n* = 15)	13	13	15
HDV-6 (*n* = 9)	9	9	9
HDV-7 (*n* = 8)	8	8	8
HDV-8 (*n* = 7)	5	7	7
Nondetermined (*n* = 2)	0	1	0

^*a*^ The Anti-HDV IgG ICT was performed with 10 µL of sera samples and the multiplex ICT was performed with 50 µL.

^*b*^ One sample from genotype HDV-4 did not have enough volume to test in the multiplex ICT.

In addition, the three assays were tested for cross-reactivity with 32 samples from other viral and non-viral hepatitis (Table S3). The ELISA exhibited nonspecific reactivity with one biliary hepatitis sample; the anti-HDV IgG ICT reacted with one hepatitis C sample, one biliary hepatitis sample, one autoimmune hepatitis sample, and two alcoholic hepatitis samples. The multiplex ICT reacted in the anti-HDV test line with four hepatitis A samples, one biliary hepatitis sample, one autoimmune hepatitis sample, and three alcoholic hepatitis samples.

## DISCUSSION

Hepatitis D remains an underdiagnosed disease, particularly in low socioeconomic regions, but also in developed countries. There is growing consensus on the need for universal testing of HDV infection in all HBsAg-positive patients (18). Therefore, broader testing is urgently needed, including for non-endemic areas (19). However, a significant gap remains in the market for commercial tests approved for HDV serodiagnosis, which is considered an essential first step in screening infected patients (18).

Here, we developed alternative recombinant proteins as antigens for the serological diagnosis of hepatitis D, derived from a consensus sequence representing the eight HDV genotypes. The final DTH10.1 protein sequence includes regions with potential B cell epitopes, based on their hydrophilicity, presence of alpha helix, and high percentage of acidic and surface-exposed amino acids (26, 27). Additionally, the DTH10.1 protein contains a high percentage of conserved epitopes for all HDV genotypes, including HDV-1, 3, and 8, which have been documented in Brazil.

The recombinant DTH10.1 and DTH10.1-SUMO were obtained as soluble proteins from *E. coli* cultures, easily purified in a single-step chromatography, and had their identity and purity confirmed by LC-MS/MS. Previously, recombinant HDAg proteins have been obtained using different expression systems, whether eukaryotic or prokaryotic (28–31). While the DTH10.1 protein was successfully applied in ELISA development, its conjugation with AuNP was unstable, probably due to its high pI value. To overcome this, DTH10.1 was fused to the SUMO tag, which enhanced protein expression and solubility and reduced its pI. The protein’s pI is an important factor for ICT development since a stable conjugation requires the AuNP at a higher pH than the pI of protein (32). Conjugation under non-ideal may lead to particle aggregation or reduced conjugation success (33, 34). Improved expression and solubility of recombinant HDAg proteins have also been reported by Ding et al. (35) who described using the thioredoxin protein as a fusion tag.

The potential of DTH10.1 for serodiagnosis was evaluated in ELISA using samples considered positive by the Diasorin ETI-AB-DELTAK-2, indicating DTH10.1 as a promising antigen for hepatitis D serodiagnosis. However, some positive samples were consistently negative in our ELISA. We used the GB HDV Ab test to confirm their status, but some discordant negative results were observed. This discrepancy may be due to a difference in time between the assays, leading to potential antibody titer loss or a false initial diagnosis. This possibility is supported by a comparative study of commercial ELISA GB HDV Ab, Dia.Pro HDV Ab and Diasorin ETI-AB-DELTAK-2, carried out by Lin et al. (36), with 1,375 samples from HBsAg-positive individuals from Taiwan where cases by HDV-2 and HDV-4 are predominant. This study showed discrepant results among the three commercial tests, but a better performance for the GB HDV Ab test.

A significant limitation of this study was the small number of positive samples with sufficient serum volume and the absence of recent results of anti-HDV antibody titers for all samples. Thus, the anti-HDV IgG ICT was initially evaluated with a 10 µL sample volume, but the sensitivity was lower than 90%. Another limitation was the lack of a similar ICT test for comparison, as no such test is available on the market. A single initiative toward obtaining a point-of-care test for hepatitis D has been described by Lempp et al. (37), which demonstrated a sensitivity of 94.6% and specificity of 100%. However, this test is not yet commercially available.

The multiplex ICT initially displayed lower sensitivity using 10 µL of sample, especially for detecting HBsAg. However, HBsAg ICTs available on the market require more than 50 µL of sample, which may explain the low sensitivity observed. In agreement, increasing the sample volume improved the test’s performance, without causing a prozone effect. Nonetheless, a slight decrease in signal intensity was observed for anti-HDV IgG antibodies detection in some samples. According to WHO criteria, the analytical sensitivity of most HBsAg ICTs ranges from 2 to 10 IU/mL; therefore, the multiplex ICT displayed a satisfactory analytical sensitivity of 5 IU/mL (38). Although several single commercial tests for HBsAg detection are available in the market, the multiplex ICT holds significant potential for use in remote and hard-to-reach endemic areas, where both types of infections are prevalent and where access to hepatitis B and D diagnostic tests is limited. It can facilitate initial screening and ongoing monitoring of these diseases in such populations.

Our results demonstrated that the three tests developed detect samples from all eight HDV genotypes with high sensitivity, showing potential as tools for diagnosing hepatitis D, with broad geographical applicability, regardless of the infecting HDV genotype. Only a few samples, mainly from genotypes 5 and 8, did not react in the tests. Comparisons of sequences of the « R0 » region used for HDV genotyping, which covers approximately half of the HDAg, showed no significant differences between the non-reactive samples and those that reacted, belonging to the same genotypes. Although variations in the unmapped regions cannot be ruled out, other factors may also contribute to these samples’ negative results. Analysis of additional samples of these genotypes may help clarify this issue.

The developed ELISA, using the DTH10.1 protein, matched other serological tests available on the market, including CLIA, a method known for high sensitivity (20, 39). It displayed 98.7% sensitivity when evaluated with samples from panel 3, which were previously characterized by CLIA. Although ELISA demands more complex laboratory conditions than rapid diagnostic tests (RDTs), it requires less specialized equipment and trained personnel than CLIA, is high throughput, can be used in automated and non-automated conditions, is highly stable, and comparably less expensive than the other available tests, making it more accessible and broadly used, even considering the laboratory structures where hepatitis D is endemic. Nonetheless, it is noteworthy that the DTH10.1 recombinant protein may be adapted as an antigen to other test formats, including CLIA.

Cross-reactivity with a few samples from other forms of hepatitis was detected with the anti-HDV test line, especially in the multiplex ICT. This may be due to the composition of these samples, which typically have high levels of liver markers, such as bilirubin, although the possibility that these patients have very low levels of antibodies that were not detected by other tests cannot be dismissed. Nonetheless, ICTs are amenable and can be adjusted to improve sensitivity and specificity. In addition, they are typically used in the context of a first step in patient screening, and the initial positive results should be confirmed by other serological or molecular tests. Further field evaluations could provide additional performance data, enabling broader validation and improvements of the reagents and prototypes developed.

In summary, the DTH10.1 recombinant proteins showed promise as antigens for diagnosing HDV infection across all genotypes. The DTH10.1-based ELISA and ICTs demonstrated high accuracy and are potential tools for improving diagnosis, regardless of the patient’s geographical origin. While there is room for further optimization, the tests offer valuable, practical, and affordable alternatives for HDV serological diagnosis. They may also prove to be effective, but still not available, tools for large-scale epidemiological surveys of hepatitis B and D in field conditions.

## Data Availability

The data from mass spectrometry analysis of DTH10.1 and DTH10.1-SUMO used can be found at https://massive.ucsd.edu/ProteoSAFe/static/massive.jsp. The login information for reviewers is “MSV000093451_reviewer” with the password “ct_vacinas_dth10”. Any other data is available from the corresponding author upon request. The GenBank accession numbers for the 47 HDAg sequences used for the full-length alignment are: AJ000558, AY633627, HM046802, KF660600, AM902173, KF660602 (Genotype 1); AJ309879, KF660599, AF104264, AF425645, AY261457, U19598 (Genotype 2); AB037947, HF679404, L22063, HF679405, LT604954, KC590319 (Genotype 3); AF018077, AF209859, AB118820, AF209859, AB118847, AB118845 (Genotype 4); AM183326, LT604958, LT604961, JX888107, LT604960, AX741159 (Genotype 5); LT604964, AM183332, LT604965, AX741164, LT604968, JX888102 (Genotype 6); LT604969, JA417541, LT604972, LT604971, SCC98322, SCC98320 (Genotype 7); AM183327, AM183330, AX741169, LT604973, LT604974 (Genotype 8).

## SUPPLEMENTAL MATERIAL

Supplemental material jcm.01999-24-s0001.pdf Figure S1 and S2, and Tables S1 to S3.

ASM does not own the copyrights to Supplemental Material that may be linked to, or accessed through, an article. The authors have granted ASM a non-exclusive, world-wide license to publish the Supplemental Material files. Please contact the corresponding author directly for reuse.
