# Supplementary material for: A universal point-of-care immunochromatographic test for the serodiagnosis of hepatitis D
Source: J Clin Microbiol. 2025 Apr 11;63(5):e01999-24. doi: 10.1128/jcm.01999-24 (PMC12077154; doi:10.1128/jcm.01999-24)
Supplement: Supplemental material — Figure S1 and S2, and Tables S1 to S3. [file jcm.01999-24-s0001.pdf]

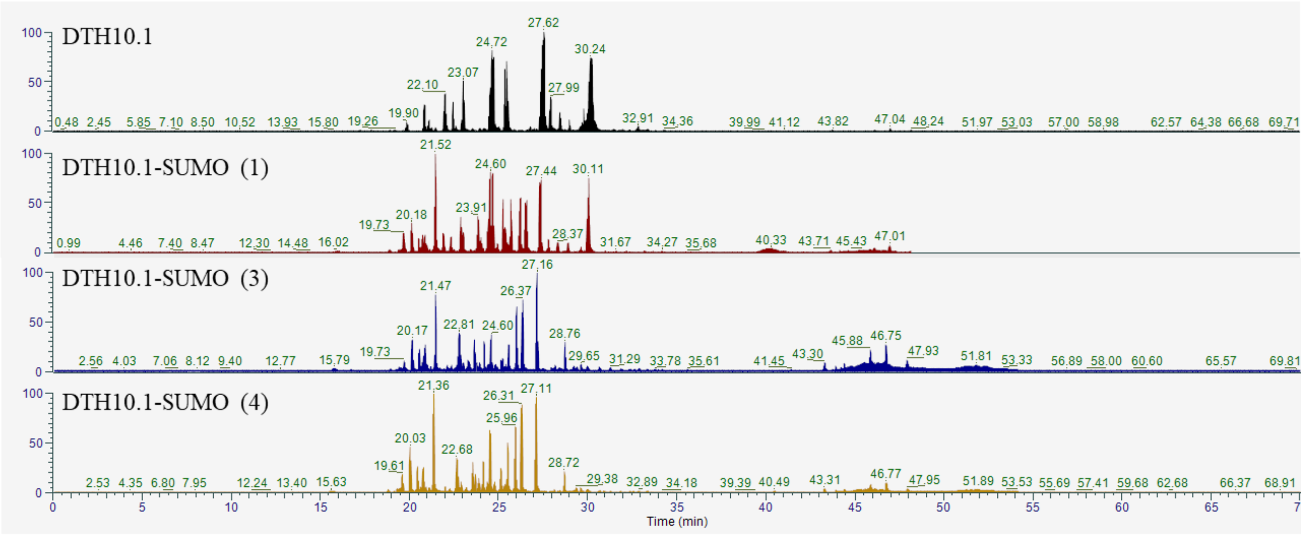

Figure S1. Mass spectra of proteins DTH10.1 and DTH10.1-SUMO. Samples 1, 3 and 4 of the DTH10.1-SUMO are the 3 protein bands observed between 25 kDa and 37 kDa.

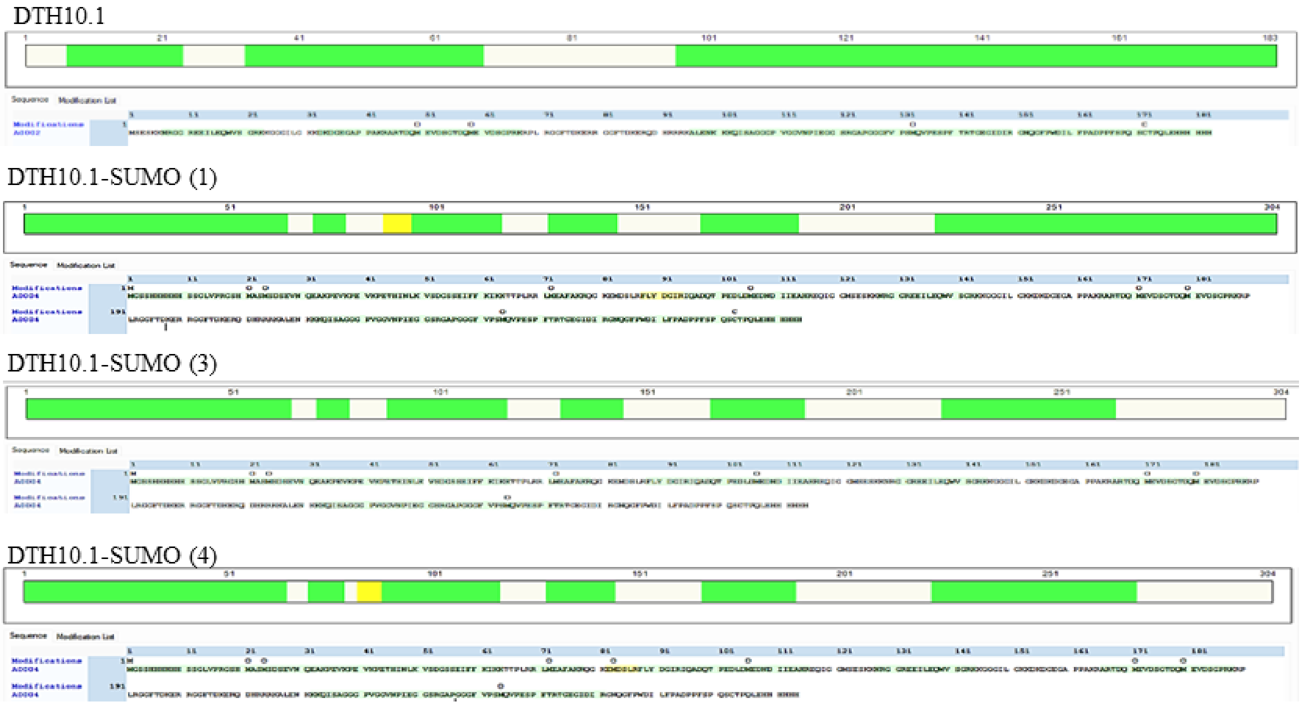

Figure S2. Analysis of the bands obtained for DTH10.1 and DTH10.1-SUMO proteins compared to the amino acid sequence of the DTH10.1 protein. The regions in green indicate the level of coverage of each sample. The coverage of DTH10.1 was 76.5% and the bands observed for DTH10.1-SUMO (1,3 and 4) had 74%, 59.5% and 65.1% of coverage, respectively.

Table S1. Average absorbance values for positive and negative samples stored at 2-8ºC and 37ºC, during the accelerated stability test.

Samples	Storage	Day 0	Day 7	Day 14	Day 21	Day 28
Negative Samples	2-8ºC	0,18	0,161	0,165	0,151	0,165
	37 ºC		0,148	0,137	0,127	0,126
Positive Samples	2-8ºC	1,80	1,696	1,685	1,637	1,710
	37 ºC		1,792	1,617	1,591	1,637

Table S2. Analytical sensitivity for detection of HBsAg of the Multiplex ICT test

Concentrations of NIBSC* Standard	Multiplex ICT HBsAg
10 UI/mL	+
9 UI/mL	+
8 UI/mL	+
7 UI/mL	S
6 UI/mL	S
5 UI/mL	S
4 UI/mL	-
3 UI/mL	-

*NIBSC standard is a HBsAg standard (HBV genotype B4, NIBSC code number 12/226) from the National Institute for Biological Standard Control (NIBSC), recommended by the WHO for this purpose.

The plus sign indicates a positive result; the S is a very weak reactivity and the minus sign is a negative result.

Table S3. Cross-reactivity analysis with samples of viral and non-viral hepatitis.

Hepatitis type	Anti-HDV ICT	Multiplex ICT[#]	ELISA
Hepatitis A (n=6)	0	4	0*
Hepatitis C (n=6)	1	0	0
Hepatitis E (n=5)	0	0	0
Biliary hepatitis (n=5)	1	1	1
Autoimmune hepatitis (n=5)	1	1	0
Alcoholic hepatitis (n=5)	2	3	0

The Anti-HDV IgG ICT was performed with 10 µL of sera samples, the Multiplex ICT with 50 µL and ELISA with 100 µL of diluted samples.

[#] The reactivities observed in the Multiplex ICT were only in the anti-HDV IgG test line.

* One sample had an indeterminate index result and was not counted as a positive result.
